# Changes of serum angiogenic factors concentrations in patients with diabetes and unstable angina pectoris

**DOI:** 10.1186/1475-2840-12-34

**Published:** 2013-02-19

**Authors:** Chun Gui, Shi-kang Li, Qin-ling Nong, Fang Du, Li-guang Zhu, Zhi-yu Zeng

**Affiliations:** 1 Department of Cardiology, the First Affiliated Hospital, Guangxi Medical University, 22 Shuangyong Road, Nanning, 530021, Guangxi, People's Republic of China

**Keywords:** Diabetes, Unstable angina pectoris, Angiogenic factors, Gensini score, Collateral vessels

## Abstract

**Backgroud:**

Diabetic microvascular changes are considered to be influenced by angiogenic factors. As a compensatory mechanism, the expression of some angiogenic factors are elevated in ischemic myocardium. The aim of this study was to investigate the changes of serum angiogenic factors, and the association among these angiogenic factors, the severity of coronary artery stenosis and collateral vessels form in patients with diabetes and unstable angina pectoris (UAP).

**Methods:**

42 patients with diabetes (diabetes group), 57 patients with UAP (UAP group), and 36 age-matched healthy people (control group) were selected. Serum concentrations of angiogenic factors were measured using cytokine array technology. The severity of coronary artery stenosis was scored using the angiographic Gensini score. Coronary collateral vessels were scored according to Rentrop's classification.

**Results:**

No significant differences in the serum concentrations of vascular endothelial growth factor (VEGF), angiopoietin-1 (Ang-1), angiopoietin-2 (Ang-2), angiogenin, angiostatin, basic fibroblast growth factor (bFGF) and platelet-derived growth factor-BB (PDGF-BB) were detected between control group and diabetes group. But in patients with diabetes complicated with UAP and in patients with UAP without diabetes, serum concentrations of VEGF and Ang-2 were elevated (p < 0.01, p < 0.01). Only serum Ang-2 concentrations were significantly correlated with Gensini score (r=0.585, p < 0.001), left ventricular end diastolic diameter (r=0.501, p < 0.001), left ventricular end systolic diameter (r=0.563, p < 0.001) and left ventricular ejection fraction (r=−0.523, p < 0.001).

**Conclusion:**

Serum concentrations of VEGF and Ang-2 were increased, and diabetes didn’t affect this increases in patients with UAP. Serum Ang-2 concentrations were correlated with the severity of coronary artery stenosis.

## Introduction

Diabetes mellitus is defined as a metabolic disorder, and is an independent risk factor for a variety of cardiovascular diseases. The causes of accelerated cardiovascular disease in diabetic patients are multifactorial, including coronary artery disease and microvascular pathology. In myocardium of diabetes, microvessels density is significantly reduced, which is reflected by a significant decrease in blood flow [1,2]. In patients with diabetes, the ability to respond to progressive coronary artery stenosis by growing coronary artery collaterals is also significantly impaired [3,4]. These microvascular changes may lead to reduced perfusion of myocardium and contribute to adverse cardiovascular events [5,6].

 Angiogenesis is a highly regulated process, requires coordinated signaling events among a variety of angiogenic factors. Diabetic microvascular complications are considered to be influenced by angiogenic factors. Vascular endothelial growth factor (VEGF), angiopoietin-1 (Ang-1) and angiopoietin-2 (Ang-2) play crucial roles in developmental blood vessel formation and regulation of hypoxia-induced tissue angiogenesis [7-9]. In addition, some other angiogenic factors and protein are also involved in angiogenesis, such as angiogenin [10], angiostatin [11], basic fibroblast growth factor (bFGF) [12] and platelet-derived growth factor-BB (PDGF-BB) [13]. Previous studies have reported that the expression of VEGF and Ang-1 were reduced, but angiostatin was elevated in the diabetic myocardium [14,15]. However, the changes of angiogenic factors expression in local tissues are not always consistent with the changes of systemic concentrations. Some studies reported that the serum VEGF concentrations were no changed in diabetes alone [16,17], but were elevated in diabetes with atherosclerosis or diabetic retinopathy [18]. As a compensatory mechanism, the expression of some angiogenic factors are elevated in ischemic myocardium [19]. Some studies showed serum concentrations and myocardial expression of VEGF and Ang-2 were increased in acute coronary syndrome [20-22].

However, little studies reported the association among circulating angiogenic factors, the severity of coronary artery stenosis and collateral vessels form in patients with diabetes and unstable angina pectoris (UAP). Therefore, using protein array technology, we examined the changes of serum angiogenic factors, including VEGF, Ang-1, Ang-2, angiogenin, angiostatin, bFGF and PDGF-BB, and the association among these angiogenic factors, the severity of coronary artery stenosis and collateral vessels form in patients with diabetes and UAP.

## Methods

### Patients recruitment and data collection

This study was approved by the Human Research Ethics Committee of the first affiliated hospital of guangxi medical university. Informed consent was obtained from all patients. 42 patients with diabetes (diabetes group) and 57 patients with UAP (UAP group) were selected for study group, and 36 age-matched healthy people from medical examination center were selected as control group. The UAP group was divided into diabetes subgroup (23 cases, UAP(DM) subgroup) and non-diabetes subgroup (34 cases, UAP(non-DM) subgroup) according to UAP with or without diabetes. UAP was defined as the presence of typical angina at rest or on minimum exertion associated with acute and transient ST-T segment ECG changes but with normal cardiac enzymes. Exclusion criteria included symptomatic peripheral vascular diseases, renal failure, liver diseases, malignancy, connective tissue diseases, evidence of ongoing infection or inflammation, significant valvular heart diseases and atrial fibrillation.

For each patient, a data sheet was completed with the patient’s name, identification number, age, gender, history of hypertension, cigarette smoking, family history of coronary artery disease, and the results of laboratory measurement. On a separate sheet, the patient’s Gensini score and collateral score were recorded by experienced angiographers.

### Blood collection

Venous blood samples were taken from the patients with diabetes and UAP on the next morning after admission, and were taken from control group at the time of medical examination. The blood samples were placed the dry tube without anticoagulant for half an hour at room temperature, and then were centrifuged at 3000 rpm for 5 min. Serum supernatant was removed, and stored at −80°C to be used in angiogenic factors level assays. Serum concentrations of angiogenic factors were measured by protein array.

### Measurement of angiogenic factors

Serum concentrations of angiogenic factors were measured using cytokine array technology (RayBiotech, Inc). Briefly, 100ul sample diluent were added into each well and incubated at room temperature for 30 min to block slides. And then 100ul standard cytokines or samples were added to each well, and incubated at room temperature for 2 h. Arrays were washed 5 times. 80ul of the detection antibody cocktail were added to each well, and incubated at room temperature for 2 h. Arrays were washed 5 times. 80ul of Cy3 equivalent dye-conjugated streptavidin were added to each well, and incubated at room temperature in dark room for 1 h. Arrays were washed 5 times. Finally, the signals were visualized through use of a laser scanner equipped with a Cy3 wavelength. Data extraction were done with the microarray analysis software.

### Measurement of coronary artery stenosis and collateral vessels

Coronary artery stenosis and collateral vessels were measured using coronary angiography. Standard angiography, with 4 views of the left coronary artery and 2 views of the right coronary artery, was used for interpretation. The severity of coronary artery stenosis was scored using the angiographic Gensini score [23]. Coronary collateral vessels were scored according to Rentrop's classification [24]. A grade of 0 was given for no visible collaterals, 1 for small side branches filled, 2 for major side branches of the main epicardial vessel filled, and 3 for main epicardial vessel filled by collaterals. The angiograms were reviewed and the severity of coronary artery stenosis and collateral vessels were scored by an experienced angiographer, and then reviewed by a separate angiographer who was blinded to the initial reading. In cases of disagreement, angiograms were reviewed by a third angiographer who was blinded to the initial 2 readings and served as an arbitrator.

### Statistical analysis

The numerical values were expressed as the mean±SD. The data were analyzed using the SPSS12.0 statistical software. Comparisons between groups were performed by one-way ANOVA with S-N-K post hoc multiple comparisons and independent-samples *T* test. The correlations among study parameters were analyzed by Pearson’s correlation test and multivariate linear regression analysis. Statistical significance was defined as p < 0.05.

## Results

### Patient characteristics and clinical data

The baseline clinical data were summarized in Table 1. No significant differences existed between groups in terms of age, gender, total cholesterol, HDL cholesterol, LDL cholesterol, Triglycerides. As expected, patients with diabetes had significantly higher levels of glycated hemoglobin (HbA1c), fasting blood glucose (FBG) and 2-h postprandial blood glucose (PBG2h) compared with nondiabetic patients (p < 0.01). In addition, compared with UAP(DM) subgroup, serum levels of HbA1c, FBG and PBG2h were significantly increased in diabetes group.

**Table 1:** Baseline clinical characteristics of all subjects

	**Control**	**Diabetes**	**UAP(DM)**	**UAP(non-DM)**	**P Value**
	**(n=36)**	**(n=42)**	**(n=23)**	**(n=34)**	
Age (years)	59.2 ± 7.5	55.92 ± 14.72	59.3 ± 8.65	60.52 ± 9.86	NS
Male, n (%)	21 (58)	23 (57)	14 (61)	22 (64)	NS
Smoking, n (%)	11 (30)	13 (31)	8 (34)	13 (38)	NS
Hypertension n (%)	0 (0)	17 (40)	14 (60)	18 (53)	< 0.01
Total cholesterol (mmol/l)	4.37 ± 1.23	4.69 ± 1.02	4.40 ± 1.12	4.62 ± 1.35	NS
HDL cholesterol (mmol/l)	1.25 ± 0.46	1.30 ± 0.40	1.32 ± 0.42	1.53 ± 0.54	NS
LDL cholesterol (mmol/l)	2.83 ± 0.96	3.07 ± 0.823	2.53 ± 1.44	2.75 ± 1.05	NS
Triglycerides (mmol/l)	1.65 ± 1.32	1.95 ± 1.50	1.89 ± 1.76	1.79 ± 1.68	NS
HbA1c (%)	-	9.09 ± 3.01	8.33 ± 3.52	5.62 ± 0.86	< 0.01
FBG (mmol/L)	5.36 ± 1.82	11.84 ± 6.67	8.3 ± 4.1	6.13 ± 1.34	< 0.01
PBG2h (mmol/L)	-	14.55 ± 7.75	11.23 ± 6.31	7.32 ± 1.51	< 0.01
Diabetes duration (year)	-	5.61 ± 2.95	6.48 ± 3.62	-	NS
Medications, n (%)					
Insulin	-	6 (14)	3 (13)	-	NS
Antidiabetics	-	19 (45)	13 (56)	-	NS
Statin	-	15 (35)	19 (83)	26 (76)	< 0.01
Aspirin	-	17 (40)	21 (91)	29 (85)	< 0.01
Clopidogrel	-	0 (0)	5 (22)	6 (18)	< 0.01
ACE inhibitor	-	11 (26)	12 (52)	16 (47)	< 0.01
Beta-receptors blocker	-	3 (7)	7 (30)	13 (38)	< 0.01
Nitroglycerin	-	0 (0)	8 (35)	13 (38)	< 0.01

In diabetes group, there were 16 patients with albuminuria, 5 patients with peripheral neuropathy. In UAP(DM) subgroup, there were 9 patients with albuminuria, 3 patients with peripheral neuropathy. There were not clinical evidence of other complications.

### Changes of serum angiogenic factor concentrations in diabetic patients

Serum concentrations of VEGF, Ang-1, Ang-2, Angiogenin, Angiostatin, bFGF and PDGF-BB were measured using cytokine array technology. The results showed there were no significant differences in the serum concentrations of all 7 angiogenic factors between control group and diabetes group. We also examined the serum concentrations of angiogenic factors in diabetic patients with UAP. The results showed the serum concentrations of VEGF and Ang-2 were significantly increased, and the serum concentrations of the other angiogenic factors were not changed in diabetic patients with UAP compared with control group (Table 2).

**Table 2:** Serum concentrations of angiogenic factors in different groups (pg/ml)

	**Control**	**Diabetes**	**UAP(DM)**	**P value**
	**(n=36)**	**(n=42)**	**(n=23)**	**(ANOVA)**
Angiogenin	4403.05 ± 850.27	4432.91 ± 1009.52	4375.56 ± 911.94	0.879
Ang-1	38647.4 ± 17964.15	39672.61 ± 14701.38	45267.17 ± 17279.20	0.205
Ang-2	2444.50 ± 1152.21	2864.76 ± 1436.20	3532.10 ± 1813.72	< 0.01
Angiostatin	7282.67 ± 2678.44	6602.22 ± 2357.75	8145.66 ± 3600.28	0.163
bFGF	890.05 ± 518.09	932.81 ± 384.60	767.36 ± 546.01	0.252
PDGF-BB	10986.56 ± 5571.85	10264.32 ± 5427.67	10737.34 ± 2851.41	0.836
VEGF	171.92 ± 106.63	239.24 ± 115.80	286.90 ± 217.01	< 0.01

### Changes of serum angiogenic factor concentrations in patients with UAP

The results showed the serum concentrations of VEGF and Ang-2 were significantly increased in UAP group compared with the control group (p < 0.01, p < 0.01). Whereas, no significant differences in the serum concentrations of angiogenin, Ang-1, angiostatin, bFGF and PDGF-BB were detected between control group and UAP group (Table 3).

**Table 3:** Serum angiogenic factors concentrations in control and UAP group (pg/ml)

	**Control**	**UAP**	**P value**
	**(n=36)**	**(n=57)**	**(*T *test)**
Angiogenin	4403.05 ± 850.27	4091.73 ± 784.89	0.105
Ang-1	38647.4 ± 17964.15	41182.45 ± 13633.35	0.456
Ang-2	2444.50 ± 1152.21	3679.91 ± 1880.94	< 0.01
Angiostatin	7282.67 ± 2678.44	7661.21 ± 3283.94	0.593
bFGF	890.05 ± 518.09	757.32 ± 544.35	0.191
PDGF-BB	10986.56 ± 5571.85	10880.50 ± 2307.80	0.941
VEGF	171.92 ± 106.63	319.35 ± 242.59	< 0.01

In order to observe the influence of diabetes on the serum concentrations of angiogenic factors in patients with UAP, we compared the serum concentrations of angiogenic factors between UAP patients with and without diabetes. The results showed there were no significant differences in the serum concentrations of all 7 angiogenic factors between UAP(DM) subgroup and UAP(non-DM) subgroup (Table 4).

**Table 4:** Serum angiogenic factors concentrations in UAP with or without diabetes subgroup (pg/ml)

	**UAP(DM)**	**UAP(non-DM)**	**P value**
	**(n=23)**	**(n=34)**	**(*T *test)**
Angiogenin	4375.56 ± 911.94	3914.33 ± 663.69	0.077
Ang-1	45267.17 ± 17279.20	38629.50 ± 10609	0.072
Ang-2	3532.10 ± 1813.72	3773.30 ± 1812.38	0.633
Angiostatin	8145.66 ± 3600.28	7358.43 ± 3153.14	0.357
bFGF	767.36 ± 546.01	748.53 ± 558.83	0.891
PDGF-BB	10737.34 ± 2851.41	10969.97 ± 1994.01	0.714
VEGF	286.90 ± 217.01	339.63 ± 262.08	0.391

### Correlation of angiogenic factors with other parameters

**Figure 1:**
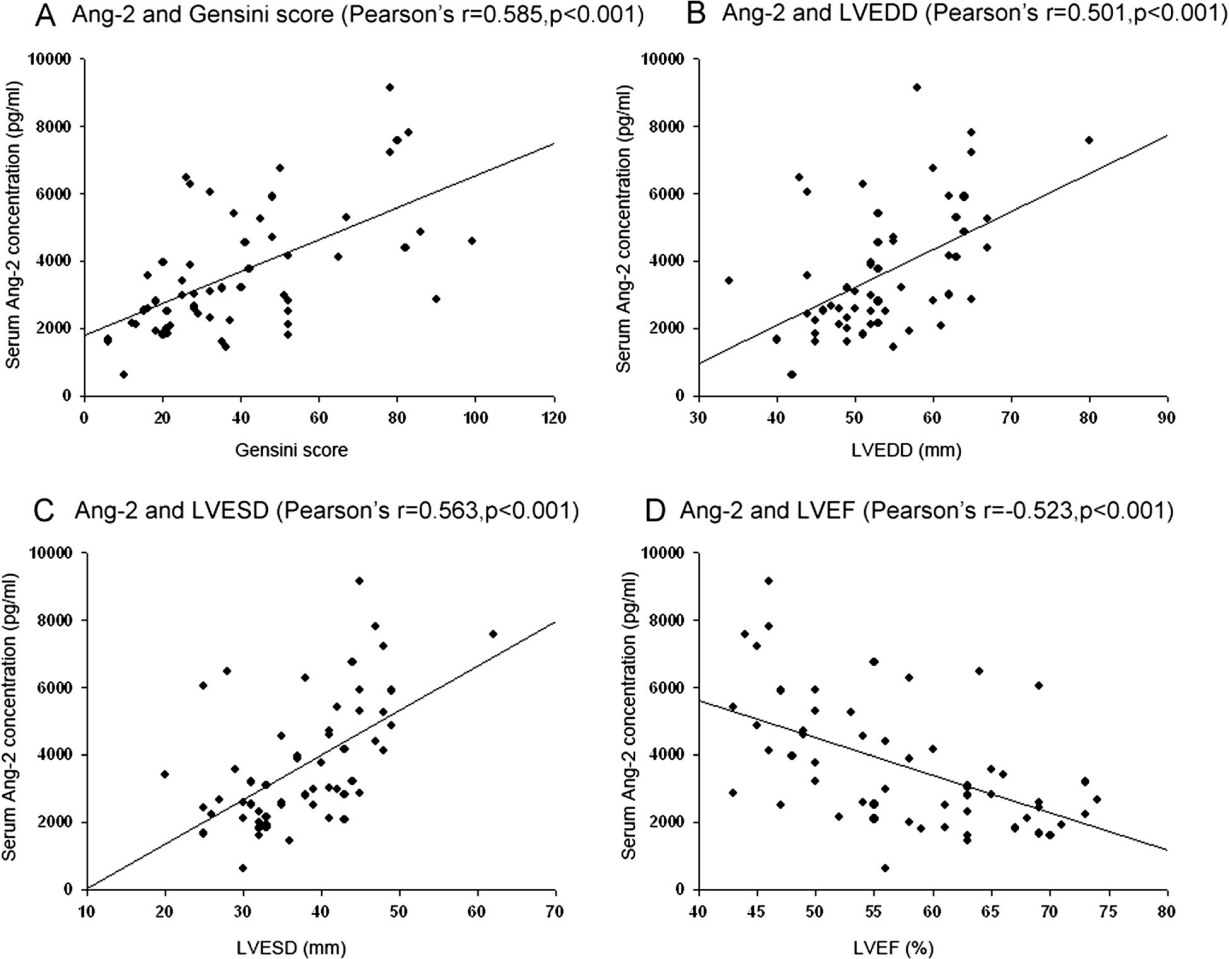
**Correlations between serum Ang-2 concentrations and Gensini score, LVEDD, LVESD and LVEF. (A)**: Ang-2 and Gensini score (Pearson’s r=0.585, p < 0.001); **(B)**: Ang-2 and LVEDD (Pearson’s r=0.501, p < 0.001); **(C)**: Ang-2 and LVESD (Pearson’s r=0.563, p < 0.001); **(D)**: Ang-2 and LVEF (Pearson’s r=−0.523, p < 0.001).

**Figure 2:**
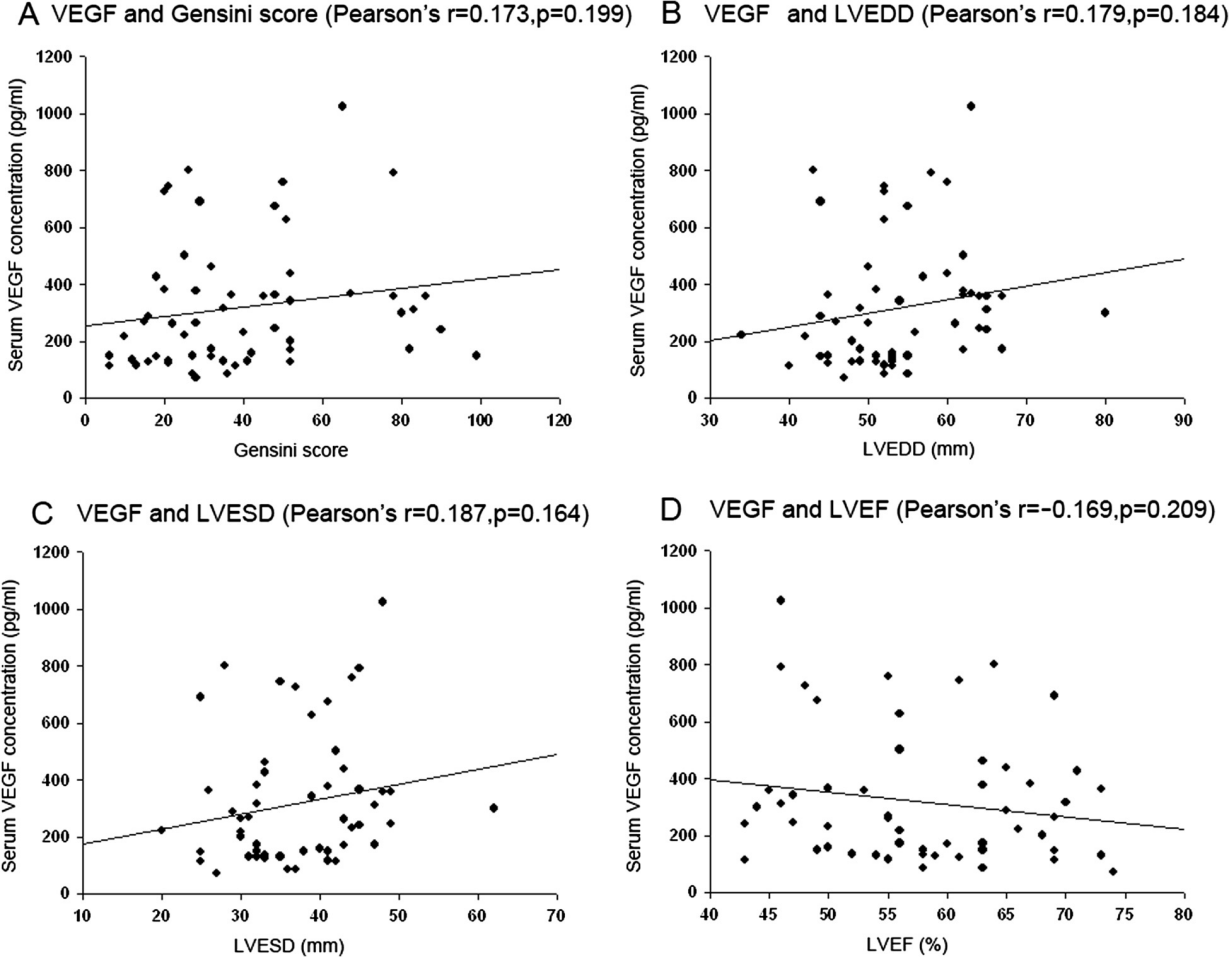
**Correlations between serum VEGF concentrations and Gensini score, LVEDD, LVESD and LVEF. (A)**: VEGF and Gensini score (Pearson’s r=0.173, p=0.199); **(B)**: VEGF and LVEDD (Pearson’s r=0.179, p=0.184); **(C)**: VEGF and LVESD (Pearson’s r=0.187, p=0.164); **(D)**: VEGF and LVEF (Pearson’s r=−0.169, p=0.209).

All 57 patients with UAP were undergone coronary angiography. The Table 5 showed the echocardiographic parameters, Gensini score, collateral score and serum NT-proBNP concentrations. By Pearson’s correlation and multivariate linear regression analysis in UAP group, serum VEGF and Ang-2 concentrations were unrelated to total cholesterol, HDL cholesterol, LDL cholesterol, triglycerides, HbA1c, FBG, PBG2h, serum NT-proBNP concentrations and collaterals score. Serum Ang-2 concentrations, but not VEGF concentrations, were significantly correlated with Gensini score, left ventricular end diastolic diameter (LVEDD), left ventricular end systolic diameter (LVESD) and left ventricular ejection fraction (LVEF) (Figure 1, Figure 2).

**Table 5:** Parameters of cardiac function and coronary artery in UAP group

	**UAP(DM)**	**UAP(non-DM)**	**P Value**
	**(n=23)**	**(n=34)**	**(*T *test)**
LVEF (%)	56.50 ± 7.95	59.09 ± 9.96	NS
LVEDD (mm)	54.76 ± 7.34	52.56 ± 9.72	NS
LVESD (mm)	38.24 ± 6.72	36.22 ± 9.65	NS
Gensini score	41.04 ± 24.52	38.59 ± 22.62	NS
Collateral score	0.78 ± 0.99	0.91 ± 1.03	NS
NT-proBNP (pg/ml)	1743.87 ± 2521.32	1513.67 ± 2102.53	NS

## Discussion

This is the first study simultaneously investigating circulating levels of 7 angiogenic factors in patients with diabetes and UAP. This study found that: (1) There were no significant differences in the serum concentrations of all 7 angiogenic factors between control group and diabetes group; (2) Serum concentrations of VEGF and Ang-2 were elevated in UAP patients with or without diabetes, and diabetes didn’t affect the elevation of serum VEGF and Ang-2 concentrations; (3) Only serum Ang-2 concentrations were significantly correlated with Gensini score, LVEDD, LVESD and LVEF.

### Changes of the serum concentrations of angiogenic factors in diabetes alone

 Many studies have demonstrated the alterations of various angiogenic factors in local tissues in the setting of diabetes. It is established that VEGF expression in diabetic myocardium is decreased [2,14,25]. Huang et al. reported that VEGF production in endothelial progenitor cell in response to high glucose stimulation was significant decreased [26]. But VEGF expression in different tissues is different in diabetes. For example, VEGF expression in the retina and renal glomeruli are increased in diabetes [14]. Circulating VEGF may not entirely reflect expression within the myocardium and endothelial cell, it also may derive from other tissues and cells. Some studies reported the serum VEGF concentrations were not changed in diabetes alone [16,17]. So this study supports the results of previous studies.

Some studies have reported the expression of Ang-1 was reduced, but angiostatin was elevated in the diabetic myocardium [15,27]. However, the changes of angiogenic factors expression in local tissue are not always consistent with the changes of serum concentrations. Lim et al. reported plasma Ang-1 was no changed in diabetes [28,29]. Siebert et al. reported the serum angiogenin levels were decreased in diabetic patients [30,31]. But another study reported serum angiogenin concentrations were increased in diabetic children [32]. We detected no significant differences in serum concentrations of all 7 angiogenic factors between control group and diabetes group. The changes of angiogenic factors expression in different tissues are different in diabetes, which may cause no differences in serum concertrations.

### Changes of the serum concentrations of angiogenic factors in UAP

Some angiogenic factors can be induced by myocardial ischemia. Matsunaqa et al. reported myocardial expression of VEGF and Ang-2 were increased in coronary heart diseases [20]. Previous studies showed serum concentrations of VEGF and Ang-2 were significantly increased, and Ang-1 were not changed in patients with acute coronary syndrome [21,22]. This study also showed serum concentrations of VEGF and Ang-2 were increased in UAP patients with or without diabetes, which were consistent with previous studies. Antonio et al. reported that plasma angiogenin levels were significantly increased in acute coronary syndromes, but not changed in stable coronary artery disease [33]. The acute coronary syndromes in Antonio’s study included 49.7% raised troponin T, which was more severe than that in our study.

Diabetes is associated with reduced expression of myocardial angiogenic factors during ischemia [34]. So this study examined whether the serum concentrations of angiogenic factors during unstable angina were influenced by diabetes. This study showed there were no significant differences in the serum concentrations of all 7 angiogenic factors between UAP patients with and without diabetes. The expression of angiogenic factors in local tissues, rather than systemic concentrations of angiogenic factors may be influenced by diabetes.

### Ang-2 but not VEGF is correlated with the coronary artery stenosis

It is well established that Ang-2 modulates endothelial cell biology and destabilizes blood vessels to facilitate angiogenesis [35]. Ang-2 promotes also VEGF induced neovascularization [36,37]. In adult humans, Ang-2 is expressed only at sites of vascular remodeling [38], so circulating levels of Ang-2 may acutely reflect the vascular regeneration and repair. However, VEGF is expressed in many cells, such as endothelial cells, macrophages, smooth muscle cells and cardiocytes. In addition, there is a tremendous interindividual variability in the degree of the hypoxic regulation of VEGF [39]. These factors may be the causes that Ang-2 but not VEGF is correlated with the coronary artery stenosis.

Chong et al. reported plasma Ang-2 and VEGF levels were increased in chronic heart failure, and Ang-2 concentrations were correlated with LVEF [40]. But the etiology of heart failure were not described in Chong’s study. Our study showed LVEDD, LVESD and LVEF were significantly correlated with Gensini score. So the interpretation may be that the severity of coronary artery stenosis is the key determinate factor for Ang-2, and the changes in heart dysfunction are merely a reflection of the consequences of stenosis severity. Further research will be needed about the association of Ang-2 and heart failure independent of underlying coronary artery stenosis.

Development of collateral vessels is triggered by the pressure gradient between the coronary arteries bed caused by an obstruction. This study found that patients with well developed collaterals had more severe coronary artery stenosis. Coronary artery stenosis severity other than serum angiogenic factors levels may mainly determine the degree of coronary collateral formation. Some studies have reported some angiogenic protein in pericardial fluid were correlated with coronary collateral growth [41,42]. However, Sherman et al. reported that the degree of coronary collateral formation was not determined by differences in systemic levels of angiogenic factors [43]. The expression of angiogenic factors in local tissues, rather than systemic levels of angiogenic factors may affect coronary collateral formation.

## Conclusions

This study showed serum concentrations of VEGF and Ang-2 were increased, and diabetes didn’t affect this increases in patients with UAP. Serum Ang-2 concentrations were correlated with the severity of coronary artery stenosis.

## Abbreviations

UAP: Unstable angina pectoris; DM: Diabetes mellitus; VEGF: Vascular endothelial growth factor; Ang-1: Angiopoietin-1; Ang-2: Angiopoietin-2; bFGF: Basic fibroblast growth factor; PDGF-BB: Platelet-derived growth factor-BB; LVEDD: Left ventricular end diastolic diameter; LVESD: Left ventricular end systolic diameter; LVEF: Left ventricular ejection fraction; HbA1c: Glycated hemoglobin; FBG: Fasting blood glucose; PBG2h: 2-h postprandial blood glucose.

## Competing interests

The authors declare that they have no competing interests.

## Authors’ contributions

ZZ contributed to the design and coordination of the study, CG performed the statistical analysis and drafted the manuscript, SL carried out patient enrollment and protein array test. All authors have read and approved the final manuscript.
